# Effects of soluble β-amyloid on the release of neurotransmitters from rat brain synaptosomes

**DOI:** 10.3389/fnagi.2014.00166

**Published:** 2014-07-15

**Authors:** Guendalina Olivero, Massimo Grilli, Jiayang Chen, Stefania Preda, Elisa Mura, Stefano Govoni, Mario Marchi

**Affiliations:** ^1^Department of Pharmacy, Section of Pharmacology and Toxicology, University of GenoaGenoa, Italy; ^2^Department of Drug Sciences, Centre of Excellence in Applied Biology, University of PaviaPavia, Italy; ^3^Center of Excellence for Biomedical Research, University of GenoaGenoa, Italy

**Keywords:** beta-amyloid, dopamine release, nicotinic receptors, muscarinic receptors, Alzheimer’s disease

## Abstract

Contradictory results have been reported on the interaction of beta-amyloid (Aβ) with cholinergic receptors. The present paper investigates the modulatory effect of Aβ1-40 on the neurotransmitter release evoked by nicotinic (nAChRs) and muscarinic (mAChRs) receptors. Aβ1-40 inhibits both nicotinic and muscarinic-evoked [^3^H]DA overflow from rat nerve endings. Added to perfusion medium, Aβ1-40 is able to enter into synaptosomes; it exerts its inhibitory effect at extracellular sites when release is stimulated by nAChRs and intracellularly when release is evoked by mAChRs. Moreover, our data show that Aβ1-40 acts as non competitive antagonist of heteromeric α4β2* but not of α3β4* nAChRs which modulate [^3^H]NA overflow. Positive allosteric modulators of nAChRs counteract its inhibitory effect. It might be that compounds of this type could be useful to prevent, slow down the appearance or reverse the cognitive decline typical of the normal processes of brain aging.

## Introduction

The predominant clinical symptoms early associated with Alzheimer’s disease (AD) include a deficiency in memory capabilities and these deficits are linked to a selective impairment of cholinergic function (Buckingham et al., 2009; Jürgensen and Ferreira, 2010). It has been reported that a significant decrease in the number of α4 nicotinic acetylcholine receptors (nAChRs) is one of the earliest events in the pathogenesis of AD (Burghaus et al., 2000) even preceding cholinergic neuronal degeneration. At this regard, accumulating evidences have shown that beta-amyloid (Aβ), which plays a crucial role both in the early and late phases of AD, disrupts cholinergic neurotransmission by interacting with presynaptic cholinergic receptors function (Cleary et al., 2005; Lesné et al., 2006; Mura et al., 2010a; Govoni et al., 2014). Indeed, several findings suggest that this interaction serves both a normal physiological process, probably regulating synaptic plasticity (Parihar and Brewer, 2010), as well as contributes to the molecular etiology of AD (Dineley, 2007; Bukanova et al., 2014 and references therein) as well as contributes to the molecular etiology of AD (Dineley, 2007 and references therein). It has to be noted that the blockade of cholinergic receptors function by Aβ could also provoke significant secondary effects if these receptors modulate neurotransmitters release. This is, for instance, the case of most nAChRs located on nerve endings, which elicit the release of several neurotransmitters in the CNS (Wonnacott, 1997).

Aβ can interact in different ways with the nAChRs depending on the receptor subtype as well as on Aβ peptide concentration or type of preparation (monomers versus oligomers) or incubation times (Jürgensen and Ferreira, 2010). It seems that Aβ might bind with high affinity (picomolar range) to α7 receptors (Wang et al., 2000; Khan et al., 2010; Tong et al., 2011), although this remains controversial (Small et al., 2007), and produces both stimulatory (Dougherty et al., 2003; Puzzo et al., 2008; Mura et al., 2012) and inhibitory effects (Liu et al., 2001; Pettit et al., 2001) suggesting a distinct regulatory role depending on the receptor location (Tong et al., 2011). Conversely, at higher concentration (nanomolar) it produces inhibitory effects (Mura et al., 2012; Zappettini et al., 2012).

Contradictory results have been also reported describing the interaction of Aβ with heteromeric nAChR subtypes. Aβ appears to bind with a lower affinity (nanomolar) to these receptors producing a functional inhibitory effect (Liu et al., 2001; Pettit et al., 2001), while similar concentrations elicit in other studies receptor activation (Pym et al., 2005). Moreover, it has been also reported that Aβ inhibits only presynaptic α7 nAChRs without any harmful effects on α4β2 nAChR subtypes (Chen et al., 2006).

Interestingly, we have previously demonstrated that Aβ inhibits preferentially the effect of stimulatory muscarinic receptors (mAChRs), leaving unchanged the function of inhibitory subtypes (Grilli et al., 2010; Mura et al., 2010b). However, there are no evidences of a direct interaction of Aβ with these receptors and consequently little is known about the Aβ inhibitory mechanism. It is also possible that the effect of Aβ on these mAChRs is indirect, although we cannot exclude the possibility that Aβ might act on an unknown site downstream the muscarinic signal (Janíčková et al., 2013).

Moreover, since the presence of intraneuronal Aβ immunoreactivity has been reported (Mucke and Selkoe, 2012 and references therein), the possibility that the early synaptic dysfunction might be generated also by the intraneuronal presence of Aβ should be considered (Oddo et al., 2006; Spires-Jones and Hyman, 2014). Indeed, several studies suggest that intraneuronal Aβ might be more dangerous for the neuron than that secreted (Lundgren et al., 2014 and references therein).

In conclusion, the interactions between Aβ and cholinergic receptors in the CNS are yet to be elucidated and the mechanism of action of the peptide is not yet fully understood.

We have thus decided: (a) to investigate the functional effects of Aβ1-40, present outside or inside the nerve endings, on the presynaptic modulation of the release of dopamine (DA) evoked by nicotinic and muscarinic agonists in rat nucleus accumbens (NAc); and (b) to explore whether selective agents can antagonize the action of Aβ.

The results indicate that Aβ1-40 inhibits both nicotinic and muscarinic cholinergic modulation of DA release acting respectively from outside and inside the nerve endings. Moreover, the inhibitory effect of Aβ1-40 on nAChRs can be antagonized by desformylflustrabromine (DFBr) and galantamine with a mechanism that presumably involves the positive allosteric site of nAChR.

## Materials and methods

### Animals and brain tissue preparation

Adult male rats (Sprague–Dawley, 200–250 g) were housed at constant temperature (22 ± 1°C) and relative humidity (50%) under a regular light–dark schedule (light 7.00 a.m.–7.00 p.m.). Food and water were freely available. The animals were killed by decapitation and the brain was rapidly removed at 0–4°C. Fresh tissue was dissected according to Paxinos and Watson (1986) sections between Bregma 0.7–2.2 mm for NAc. Experiments were approved by the Ethical Committee of the Pharmacology and Toxicology Section, Department of Pharmacy, in accordance with the European legislation (2010/63/EU) and were approved by Italian legislation on animal experimentation (protocol number 124/2003-A). All efforts were made to minimize animal suffering and to use the minimal number of animals necessary to produce reliable results.

### Preparation of β amyloid solutions

For our experiments, synthetic human Aβ1-40 was dissolved in a CSF at a concentration of 100 μM (stock solution). Then, this solution was filtered through a Millipore 0.2 μm pore membrane and stocked in small aliquots at −80°C. Working solutions were freshly prepared by diluting an aliquot of Aβ1-40 stock solution at the final concentrations used for *in vitro* analysis just before the administration.

As far as the characteristics of the Aβ peptides we employed in our study, we have previously shown that in our experimental conditions the solutions of Aβ are mostly formed by Aβ monomers. However, we cannot completely exclude that small amounts of Aβ oligomers are also present in light of the fact that aggregation is a concentration and time-dependent process (Mura et al., 2012).

### Release experiments from synaptosomes

Crude synaptosomes from rat NAc or hippocampus were prepared according to Grilli et al. (2010).

In release experiments, NAc synaptosomes were incubated for 20 min at 37°C with [^3^H]dopamine ([^3^H]DA; final concentration 0.03 μM) in the presence of 6-nitroquipazine (final concentration 0.1 μM), to avoid false labeling of serotonergic terminals and of desipramine (final concentration 0.1 μM), to avoid false labeling of noradrenergic terminals. In a set of experiments, hippocampal synaptosomes were incubated with [^3^H]noradrenaline ([^3^H]NA; final concentration 0.03 μM) in the presence of 6-nitroquipazine.

Identical portions of the synaptosomal suspension were then layered on microporous filters at the bottom of parallel superfusion chambers thermostated at 37°C (Patti et al., 2006; Grilli et al., 2008). Synaptosomes were superfused at 0.5 ml/min with standard physiological medium. Starting from *t* = 36 min to *t* = 48 min of superfusion four consecutive 3-min fractions (b1–b4) were collected. Synaptosomes were exposed to agonists or to depolarizing agent (4-aminopyridine) at *t* = 39 min, till the end of superfusion, while other drugs (antagonists, peptides, antibody, allosteric modulators) were added 8 min before agonists.

To introduce into synaptosomes a specific amount of Aβ1-40 or 6E10 monoclonal antibody we used an experimental approach previously experienced (Raiteri et al., 2000; Grilli et al., 2012). When indicated, rat NAc was homogenized in buffered sucrose containing Aβ1-40 (2 μM, 200 nM, 20 nM and 2 nM) or 6E10 monoclonal antibody (1 mM) in order to entrap these agents into subsequently isolated synaptosomes. Based on estimates made by entrapping of [^3^H]sucrose, the intrasynaptosomal concentration of the compounds is about 5% of the original concentration in the homogenization medium (Raiteri et al., 2000).

Samples collected and superfused synaptosomes were then counted for radioactivity (fractional efflux). Agonist-induced effect was expressed as % induced overflow and was evaluated by subtracting the neurotransmitter content released in the four fractions collected under basal condition (no drug added) from that released in presence of the stimulus.

### Dot blot analysis

Dot blot analysis of synaptosomal content of Aβ1-40 and 6E10 monoclonal antibody was performed according to De Felice et al. (2007), with slight modifications.

NAc synaptosomes were incubated in physiological medium with Aβ1-40 (100 nM) for 10 min at 37°C, in order to evaluate the entry of Aβ into nerve terminals under the same conditions of release experiments. Synaptosomes were precipitated, washed in phosphate-buffered saline (PBS) and lysed in cold water with sonication. Samples were then centrifuged at 29000 g for 30 min in order to precipitate membranes. The supernatant (cytosolic fraction) was spotted on a nitrocellulose membrane (5 μg of synaptosomal proteins for each sample). Membrane was incubated for 1 h at room temperature in Tris-buffered saline-Tween (t-TBS: 0.02 M Tris, 0.150 M NaCl, and 0.05% Tween 20) containing 5% non-fat dried milk, in order to block non-specific binding sites. Then, it was incubated with 6E10 monoclonal antibody (1:2000 in milk/t-TBS 5%), washed three times in t-TBS and incubated with horseradish peroxidase-linked anti-mouse secondary antibody (1:10000 in milk/t-TBS 5%) for 1 h at room temperature. Immunoblots were visualized with an ECL (enhanced chemiluminescence) Plus Western blotting detection system to detect protein spots. The same procedure was used to evaluate the presence of Aβ1-40 or 6E10 antibody in entrapped synaptosomes at the end of release experiments (40 min). When we studied 6E10 antibody entrapped synaptosomes, membrane was incubated only with anti-mouse secondary antibody (1:10000 in milk/t-TBS 5%) for 1 h at room temperature.

All the nitrocellulose membranes were finally treated with Ponceau Red solution to verify the presence of synaptosomal proteins.

### Statistical analysis

Multiple comparisons were performed with one- or two-way ANOVA followed by the Bonferroni *post-hoc* test. Data were considered significant for *P* < 0.05, at least. The EC_50_ and Hill slope have been calculated according to a four parameter logistic curve using Sigma Plot (Jandel Scientific, San Rafael, CA, USA).

### Chemicals

[7,8-^3^H]Dopamine (21.2 Ci/mmol (0.784 TBq/mmol)) and [1-7,8-^3^H]noradrenaline (12.1 Ci/mmol (0.448 TBq/mmol)) were purchased from Perkin Elmer, Monza, Italy. Nicotine hydrogen tartrate salt, choline, 4-aminopyridine, 6-nitroquipazine, desipramine, beta-amyloid (40-1), horseradish peroxidase-conjugated anti-mouse secondary antibody, Ponceau Red were from Sigma (Sigma-Aldrich, St Louis, MO, USA); Monoclonal antibody 6E10 was from Covance ImmunoTechnologies, Dedham, MA; 5-iodo-A-85380, dihydro-β-erythroidine, α-conotoxin MII, oxotremorine sesquifumarate, desformylflustrabromine hydrochloride, galantamine hydrobromide, SR 165845, beta-amyloid (1-40) were from Tocris (Tocris Bioscience, Bristol, UK).

## Results

**Figure 1:**
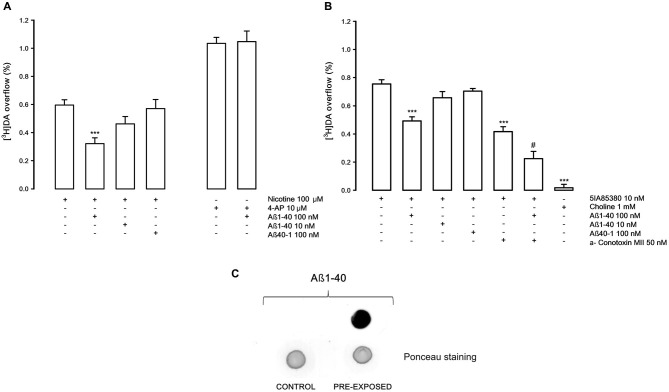
**(A)** Effects of β-amyloid (Aβ) 1-40 on nicotine and 4-aminopyridine (4-AP)-evoked [^3^H]DA overflow from rat NAc synaptosomes. Data are means ± SEM of at least four experiments run in triplicate, *** *P* < 0.001 versus nicotine. One-way ANOVA followed by Bonferroni *post-hoc* test. **(B)** Effects of Aβ1-40 on [^3^H]DA overflow evoked by 5IA85380, alone or in presence of α-conotoxin MII. Data are means ± SEM of at least four experiments run in triplicate, *** *P* < 0.001 versus 5IA85380, ^#^ *P* < 0.05 versus 5IA85380 + α-conotoxin MII. One-way ANOVA followed by Bonferroni *post-hoc* test. **(C)** Representative dot blot of rat NAc nerve endings: untreated synaptosomes (CONTROL) and synaptosomes exposed for 10 min to Aβ1-40 (PRE-EXPOSED).

Figure 1A shows that the stimulatory effect of the *in vitro* administration of nicotine on [^3^H]DA overflow, confirming previously results (Preda et al., 2008), is strongly inhibited by Aβ1-40 at 100 nM (−46%) but not at 10 nM concentration; Aβ 40-1 at 100 nM concentration is ineffective on the nicotine-evoked [^3^H]DA overflow. The [^3^H]DA overflow evoked by 4-AP (10 μM) is not altered by Aβ1-40 (100 nM). Figure 1B shows that [^3^H]DA overflow induced by 5IA85380 an α4, α6 nAChRs agonist, is inhibited by Aβ1-40 (100 nM) to a similar extent (−36%). In presence of α-conotoxin MII (50 nM), a specific inhibitor of α6 nAChRs, the 5IA85380-evoked [^3^H]DA overflow is significantly decreased (−44%) and further inhibited (−45%) by Aβ1-40 indicating that α-conotoxin MII—resistant nAChRs (α4- non α6) are inhibited by Aβ1-40. As expected, choline (1 mM) doesn’t elicit [^3^H]DA overflow from NAc synaptosomes, which is mostly modulated by non α7 nAChRs subtypes (Gotti et al., 2009 and references therein). Figure 1C reports an example of immunoblot analysis of Aß 1-40 which shows that Aß peptide, administered in the extracellular medium, enters and is present inside the isolated nerve terminals under the conditions in which the release experiments have been performed.

**Figure 2:**
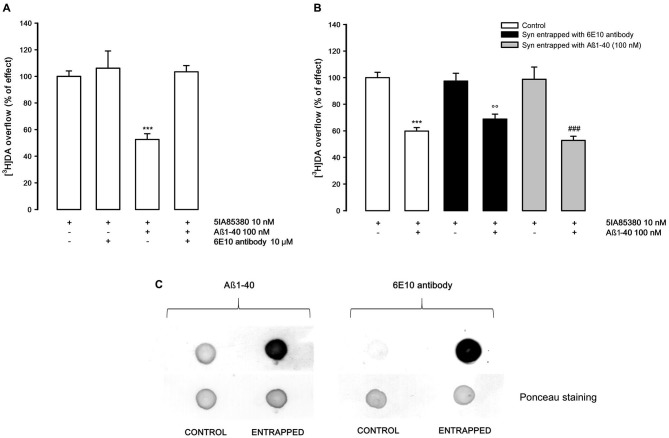
**(A)** Effect of 6E10 monoclonal antibody on the inhibition by Aβ1-40 of 5IA85380-evoked [^3^H]DA overflow from rat NAc synaptosomes. Data are means ± SEM of four experiments run in triplicate, *** *P* < 0.001 versus 5IA85380. One-way ANOVA followed by Bonferroni *post-hoc* test. **(B)** Effects of Aβ1-40 added to the medium on 5IA85380-evoked [^3^H]DA overflow from rat NAc synaptosomes entrapped with 6E10 antibody or Aβ1-40. Data are means ± SEM of at least four experiments run in triplicate, *** *P* < 0.001 versus 5IA85380 (control synaptosomes), °° *P* < 0.01 versus 5IA85380 (6E10 antibody entrapped); ^###^ *P* < 0.001 versus 5IA85380 (Aβ1-40 entrapped). Two-way ANOVA followed by Bonferroni *post-hoc* test. **(C)** Representative dot blot of rat NAc nerve endings: untreated synaptosomes (CONTROL), Aβ1-40 entrapped and 6E10 antibody entrapped synaptosomes after 40 min of incubation (ENTRAPPED).

In order to study if the Aβ1-40 inhibitory effect on nAChRs is exerted on external or internal synaptosomal targets, we investigated whether a specific Aβ antibody present in the medium or entrapped into synaptosomes may counteract the inhibitory effect of Aβ1-40. As shown in Figure 2A, the presence of Aβ antibody in the superfusion medium doesn’t modify the 5IA85380-evoked overflow of [^3^H]DA, but completely counteracts the inhibitory effect of Aβ1-40 (100 nM). Conversely, when the Aβ antibody is entrapped into synaptosomes (Figure 2B), Aβ1-40 is yet able to inhibit the 5IA85380-evoked [^3^H]DA overflow. All together these findings suggest the Aβ1-40 inhibitory effect versus the nicotinic stimulation of DA overflow is due primarily to an external modulation by Aβ1-40. This hypothesis is also confirmed by the lack of inhibitory effect of Aβ1-40 when it is entrapped into synaptosomes. As expected, in these experimental conditions Aβ1-40 added to the superfusion medium is still able to inhibit the 5IA85380 evoked [^3^H]DA overflow (Figure 2B). Figure 2C confirms the presence of 1-40 and Aβ 6E10 antibody in the entrapped synaptosomes after 40 min of superfusion.

**Figure 3:**
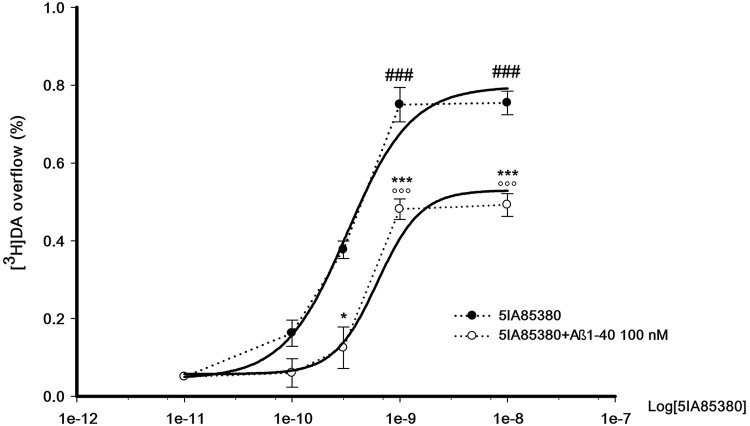
**Concentration-dependent effect of 5IA85380, alone or in presence of Abeta1-40, on [^3^H]DA overflow from rat NAc synaptosomes.** Data are means ± SEM of at least four experiments run in triplicate, ^###^ *P* < 0.001 versus 5IA85380 (100 pM), °°° *P* < 0.001 versus 5IA85380 (100 pM) + Aβ1-40 (100 nM), * *P* < 0.05 versus 5IA85380 (300 pM), *** *P* < 0.001 versus 5IA85380 (1 nM) and 5IA85380 (10 nM) respectively. One-way ANOVA followed by Bonferroni *post-hoc* test.

Figure 3 shows the concentration-dependent stimulatory effect of 5IA85380 on [^3^H]DA overflow in absence or in presence of Aβ1-40 in the perfusion medium. The EC_50_ of 5IA85380 are similar (respectively 0.32 ± 0.08 and 0.5 ± 0.12 nM) indicating a typical non-competitive antagonistic effect.

The possibility of rescuing the inhibitory effect of Aβ was investigated using two α4β2 nicotinic compounds, DFBr and galantamine, supposed to act as positive allosteric modulators at the α4β2 nAChRs (Kim et al., 2007; Pandya and Yakel, 2011).

**Figure 4:**
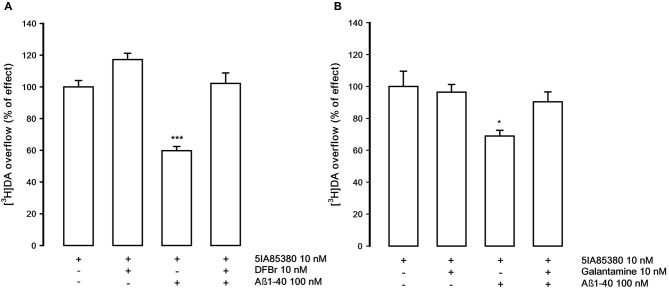
**(A)** Effect of desformylflustrabromine (DFBr) on the inhibition by Aβ1-40 of 5IA85380-evoked [^3^H]DA overflow from rat NAc synaptosomes. Data are means ± SEM of six experiments run in triplicate, *** *P* < 0.001 versus 5IA85380. One-way ANOVA followed by Bonferroni *post-hoc* test. **(B)** Effect of galantamine on the inhibition by Aβ1-40 of 5IA85380-evoked [^3^H]DA overflow from rat NAc synaptosomes. Data are means ± SEM of four experiments run in triplicate, * *P* < 0.05 versus 5IA85380. One-way ANOVA followed by Bonferroni *post-hoc* test.

Figure 4A shows that DFBr (10 nM), in our experimental conditions, is able to produce a modest, not significant, increase of the 5IA85380-evoked [^3^H]DA overflow but is able to counteract the inhibitory effect of Aβ1-40 (100 nM). Similarly, galantamine (10 nM), which is ineffective on the 5IA85380-evoked [^3^H]DA overflow, also shows the capacity of antagonizing the inhibitory effects of Aβ1-40 (Figure 4B).

**Figure 5:**
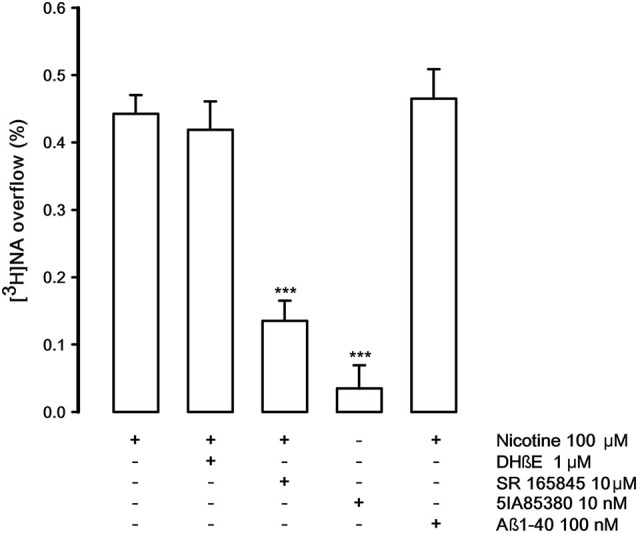
**Effect of dihydro-beta-erythroidine (DHbetaE), SR 165845 and Abeta1-40 on nicotinic-evoked [^3^H]NA overflow from rat hippocampal synaptosomes.** Data are means ± SEM of at least four experiments run in triplicate, *** *P* < 0.001 versus nicotine. One-way ANOVA followed by Bonferroni *post-hoc* test.

Figure 5 describes the pharmacological features of the nAChR that modulates the release of noradrenaline (NA) in rat hippocampus. This receptor is known to be pharmacologically different from those present on DA nerve terminals (Risso et al., 2004; Azam and McIntosh, 2006; Kennett et al., 2012). The nicotine-evoked [^3^H]NA overflow is not antagonized by DHβE (1 μM) but counteracted by the specific α3β4 antagonist SR165845 (10 μM; Zaveri et al., 2010). As expected 5IA85380 is unable to elicit [^3^H]NA overflow. Quite interestingly, Aβ1-40, at the same concentration used in the previous experiments (100 nM), doesn’t produce any inhibitory effect.

**Figure 6:**
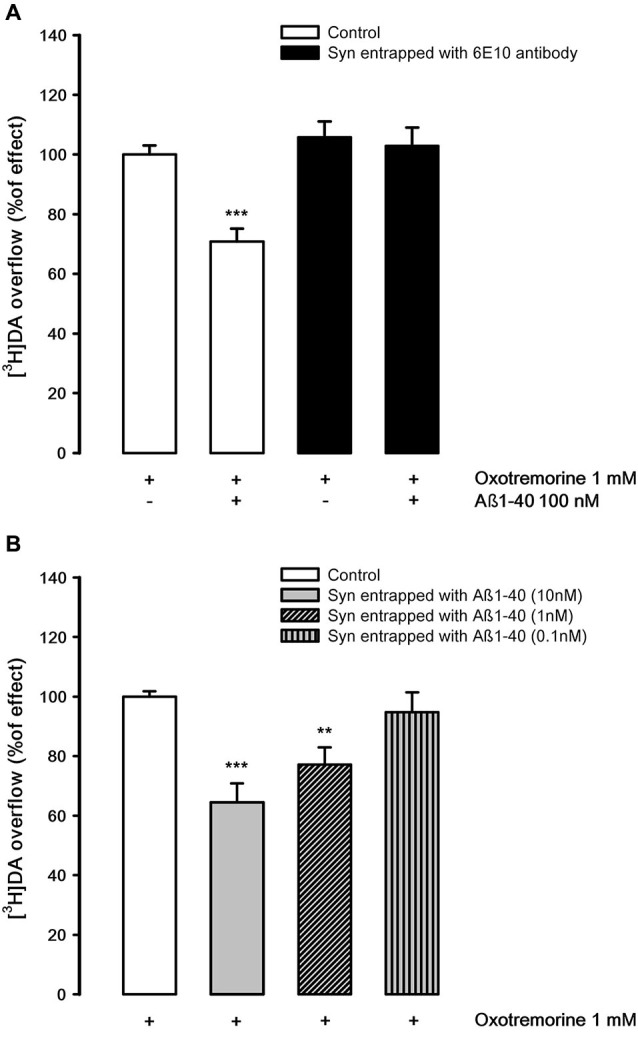
**(A)** Effects of Aβ1-40 added to the medium on oxotremorine-evoked [^3^H]DA overflow from rat NAc synaptosomes entrapped with 6E10 monoclonal antibody. Data are means ± SEM of three experiments run in triplicate. *** *P* < 0.001 versus oxotremorine (control synaptosomes). Two-way ANOVA followed by Bonferroni *post-hoc* test. **(B)** Effects of Aβ1-40 entrapped in rat NAc synaptosomes on oxotremorine-evoked [^3^H]DA overflow. Data are means ± SEM of three experiments run in triplicate. * *P* < 0.05; ** *P* < 0.01; *** *P* < 0.001 versus oxotremorine (control synaptosomes). One-way ANOVA followed by Bonferroni *post-hoc* test.

The possibility that Aβ1-40 may differentially inhibit the nAChR and mAChR subtypes which control [^3^H]DA release has also been investigated. Figure 6A shows that oxotremorine, a selective muscarinic agonist, is able to elicit [^3^H]DA overflow, probably activating an M5 mAChR subtype present on DA nerve endings (Grilli et al., 2008). Aβ1-40 (100 nM, but not 10 nM) present in the superfusion medium produces a significant inhibitory effect on M5-evoked [^3^H]DA overflow as previously demonstrated (Grilli et al., 2010) but, quite interestingly, in synaptosomes entrapped with 6E10 antibody, which doesn’t alter oxotremorine-elicited [^3^H]DA overflow, Aβ1-40 added to the superfusion medium is ineffective. These results suggest that Aβ1-40 must enter into nerve endings to produce the inhibitory effect. This has been confirmed in synaptosomes in which Aβ1-40 has been entrapped. The peptide is still capable of inhibiting the muscarinic stimulation of DA overflow at very low concentrations (10 nM and 1 nM, Figure 6B).

## Discussion

It has been reported that the extracellular application of Aβ might induce extensive neuronal changes including altered synaptic plasticity and neuronal damage. This is in agreement with experiments using monoclonal antibodies showing that the principal location of Aβ in Alzheimer brains is mostly extracellular (Mucke and Selkoe, 2012 and references therein). However, it is well accepted that Aβ in the brain is present both extracellularly and intracellularly (Mohamed and Posse de Chaves, 2011; Mucke and Selkoe, 2012). The intraneuronal Aβ peptide could represent both an amount of normally produced Aβ ready to be secreted or previously secreted Aβ monomers which have been taken back up into neurons (Walsh et al., 2000). There is now increasing evidence that intracellular Aβ accumulation is associated with neuritic and synaptic pathology (Mohamed and Posse de Chaves, 2011), in particular with early synaptic dysfunction (Oddo et al., 2006). At this regard, in triple transgenic mouse model of AD the intraneuronal presence of Aβ correlates very well with long-term potentiation abnormalities and synaptic dysfunction (Mohamed and Posse de Chaves, 2011 and references therein).

The present research shows that in rat NAc synaptosomes the extracellular application of Aβ1-40 inhibits both nicotinic and muscarinic cholinergic modulation of DA release by acting respectively from outside and inside the nerve endings likely through two different mechanisms of action.

As shown in Figure 1, Aβ1-40, used at nanomolar concentrations, is able to inhibit the overflow of [^3^H]DA elicited by the activation of nAChR subtypes which modulate the release of DA in NAc (Gotti et al., 2009 and references therein). The inhibition of nicotinic-evoked [^3^H]DA overflow depends on the interaction of Aβ1-40 with nAChRs through a non-competitive antagonism; indeed, Aβ1-40 inhibits by 40% the effect of the nicotinic agonist 5IA85380 without producing changes of its EC_50_.

The inhibitory effect of Aβ1-40 is evident both when the nAChR subtypes are stimulated by nicotine, 5IA85830 alone and in the presence of α-conotoxin MII, which excludes the involvement of the nAChRs containing the α6 subunit (likely α6β3β2 and/or α6α4β3β2). Since the inhibitory effect of Aβ against the 5IA85380-evoked [^3^H]-DA overflow is similar in the presence or in the absence of α-conotoxin MII, it can be speculated that the inhibitory effect of Aβ1-40 concerns all the α4β2* nAChR subtypes that modulate [^3^H]DA overflow including the α-conotoxin MII resistant nAChRs (probably α4β2 and/or α4α5β2). When defining the receptor subtypes we use asterisks to indicate the possible presence of unidentified nAChR subunits. Conversely, Aβ is not effective on the α3β4 nAChRs that elicit [^3^H] NA overflow in the hippocampus. As shown in Figure 5, these receptors are not inhibited by Aβ1-40 when used at the same nanomolar range of concentration active in NAc. This difference is in agreement with other previously reported findings (Pym et al., 2005). Our results seem therefore to indicate a certain specificity of action of Aβ1-40 for some selective nAChRs subtypes, although the possibility that it might be effective also on α3β4 nAChRs, when used at higher concentrations, has to be taken into consideration (Nery et al., 2013).

The nicotinic-evoked DA release is of exocytotic type involving the activation of α4β2* receptors present on the DA terminals, a subsequent depolarization of the nerve endings and the activation of voltage-operated calcium channels (Wonnacott, 1997). It is worth noting that the mechanism of DA release triggered by non-α7 nAChRs present on nerve terminals involves the activation of intracellular pathways that are not impaired by the presence of Aβ1-40, as illustrated in Figure 2B. This result clearly demonstrates that the inhibitory effect of Aβ1-40 on the function of nAChRs occurs through a mechanism external to the nerve ending. This point is further supported by the finding that Aβ1-40 is ineffective in inhibiting the depolarization-evoked DA overflow elicited by other depolarizing agents (Figure 1A).

Another significant point of this study is the demonstration that the inhibitory effect of Aβ1-40 can be reversed by the Aβ directed antibody and by some compounds acting as allosteric modulators at nAChRs. It is significant to note that the effect of Aβ1-40 is no longer evident in the presence of both DFBr and galantamine. DFBr has been characterized as selective positive allosteric modulator of α4β2* nAChRs, at low micromolar concentration, but not active on α7 or α3β4 nAChR subtypes (Weltzin and Schulte, 2010; Pandya and Yakel, 2011), while galantamine is a well-known α7 positive allosteric modulator which is also effective on α4β2 nAChRs (Samochocki et al., 2003). In our experimental conditions, DFBr (10 nM) produces only a slight increase, although not significant, of the 5IA85380-evoked DA overflow while galantamine is completely ineffective. However, both drugs are able to rescue the Aβ1-40-induced inhibition of nAChRs stimulation. In line with these findings, it has been previously shown that DFBr prevents the functional blockade by Aβ1–42 of ACh-induced currents in oocytes expressing α4β2 receptors (Pandya and Yakel, 2011). From our results, it is difficult to establish the precise mechanism of action of the two drugs. However, since it is inconceivable to assume that the Aβ1-40 inhibitory effect might be compensated by a functional potentiating effect of these agents if administered together with Aβ1-40 it is therefore reasonable to speculate that the antagonistic effect of the two drugs might be due to a direct interference with the site of action of Aβ1-40. Whether this relates to the allosteric site of the α4β2* nAChR or to other binding sites is difficult to determine. Nonetheless, it is interesting to mention that drugs acting as positive allosteric modulators on α4β2* do not work as modulators on α3β4 nAChRs (Williams et al., 2011) and Aβ1-40 is ineffective on this nAChR subtype.

A different view emerged when considering the muscarinic control of DA release in the same brain area. In a recent study, we pharmacologically characterized as M5 the mAChR subtype which modulate DA release in the rat NAc and reported that it can be inhibited by the extracellular application of Aβ1-40 at nM concentration (Grilli et al., 2010). Here we clearly show that the inhibitory effect of Aβ1-40 on the mAChR-elicited [^3^H]DA overflow is not due to the binding of Aβ1-40 to the external surface of mAChRs or to binding to other external sites. Indeed, this inhibitory effect is achieved inside the nerve terminal through a mechanism which possibly requires the binding of Aβ1-40 to a site downstream the mAChR signal (Figures 6A,B). This finding was in a way expected since data from the literature confirm that Aβ1-40 does not bind to any mAChRs (Kelly et al., 1996; Wang et al., 2000). Quite interestingly, Aβ1-40 present in the superfusion medium is therefore able to enter into the nerve endings (cf. Figure 1C) and to perform its selective inhibitory action from inside. It is interesting that Aβ1-40 present inside the nerve endings inhibits the muscarinic stimulation of [^3^H]DA release in concentrations much lower (1 nM) than those effective in interfering with the nicotinic modulation outside the nerve endings (100 nM; cf. Figures 6B, 1A). The fact that Aβ1-40 is effective inside the neuron at very low concentrations could be relevant suggesting that Aβ1-40 could be more likely connected with the small production from APP that occurs inside the neuron (Mohamed and Posse de Chaves, 2011) and not necessarily due to the entry of Aβ from the extracellular environment. This should be a further support to the hypothesis that the effect of Aβ at the intracellular level might be an earlier pathological event which precedes other neurodegenerative processes.

It is well accepted that the facilitation of neurotransmitter release by M1, M3 and M5 mAChRs is mediated by signal transduction pathways involving the protein kinase C (Zhong et al., 2003). Therefore the possibility that Aβ1-40 may act inhibiting this function downstream the mAChR activation is likely to occur (Olariu et al., 2002; Zhong et al., 2003; Preda et al., 2008; Mura et al., 2010a), although other mechanisms of action cannot be excluded.

## Conclusion

In conclusion, we have demonstrated that Aβ1-40 inhibits both nicotinic- and muscarinic-evoked DA overflow by two different mechanisms: interacting extracellularly with a nAChRs binding site or through an internal modulation involving the downstream signal transduction of M5 mAChR subtypes. Moreover, we have found that the inhibitory effect of Aβ1-40 on nAChRs could be rescued by DFBr and galantamine with a mechanism which probably involved the positive allosteric modulator site of α4*nAChRs.

The demonstration that the inhibitory effect of Aβ can be selectively reverted may open up new and important therapeutic perspectives, especially with regard to the potential use of drugs of this type in the early stages of the disease when it could be very important to slow down its progression. As reported in the introduction, a variety of studies indicates that Aβ may have an important role in cognitive dysfunction and memory deficits. It might be that compounds of this type could be useful even in healthy subjects to prevent, slow down the appearance or reverse the cognitive decline typical of the normal processes of brain aging.

## Author contributions

Guendalina Olivero performed the release and the dot-blot experiments, revised critically the paper and approved the final version; Jiayang Chen, Stefania Preda, and Elisa Mura performed the release experiments and revised critically the paper and approved the final version; Massimo Grilli contributed to the design of the work and coordinated the release experiments, performed the dot-blot experiments, revised critically the paper and approved the final version; Stefano Govoni and Mario Marchi provided a substantial contributions to the design of the work and to the interpretation of data and wrote the paper.

## Conflict of interest statement

The authors declare that the research was conducted in the absence of any commercial or financial relationships that could be construed as a potential conflict of interest.
